# Experience over tools: does the use of magnifying loupes reduce the incidence of transient postoperative hypocalcemia after total thyroidectomy with central neck dissection? – a single-centre prospective randomized study

**DOI:** 10.3389/fendo.2026.1811572

**Published:** 2026-05-20

**Authors:** Wojciech Sielski, Tomasz Osęka, Artur Antolak, Łukasz Kaska, Anton Żawrocki, Michał T. Kucewicz, Dariusz Dymecki

**Affiliations:** 1 Department of General Surgery, Ministry of the Interior and Administration Hospital, Gdańsk, Poland; 2 Alab Plus Sp. z o.o., Pathology, Gdańsk, Poland; 3 Departament of Pathology, Specialist Hospital in Wejherowo, Wejherowo, Poland; 4 BioTechMed Center, Multimedia Systems Department, Faculty of Electronics, Telecommunications and Informatics, Gdansk University of Technology, Gdańsk, Poland

**Keywords:** central neck dissection, hypoparathyroidism, loupe magnification, magnifying loupes, papillary thyroid carcinoma, parathyroid preservation, surgical training, thyroidectomy

## Abstract

**Background:**

Parathyroid preservation during total thyroidectomy with central neck dissection in papillary thyroid carcinoma remains challenging due to the glands’ delicate vascular anatomy and risk of inadvertent devascularization or excision. Although advanced technologies exist, lowcost 2.5× magnifying loupes have been proposed as a simple alternative. High-level evidence of their benefit in experienced hands is limited.

**Methods:**

This single-centre prospective randomized controlled trial enrolled 108 patients with low-risk papillary thyroid carcinoma (T1–T2, single tumour ≤2 cm) undergoing total thyroidectomy plus prophylactic level VI central neck dissection. Patients were randomized 1:1 to the magnifying loupes group (ML T+6L, n=54) or naked-eye control group (T+6L, n=54). All procedures were performed by the same experienced surgeon using a standardized capsular dissection technique. The primary endpoint was the incidence of transient postoperative hypocalcemia (serum calcium <8.0 mg/dL on postoperative day 2 and/or need for calcium supplementation).

**Results:**

Transient hypocalcemia occurred in 31.5% of patients in the loupes group versus 33.3% in the control group (p=1.000). There were no significant differences in clinical symptoms of hypocalcemia, need for intravenous calcium, change in serum calcium (ΔCa), change in PTH (ΔPTH), number of identified parathyroid glands, or rate of incidental parathyroid excision. Operative time was significantly longer with loupes (144.1 ± 31.7 vs 125.4 ± 29.8 min; p=0.001). No cases of permanent hypoparathyroidism occurred in either group during 6-month follow-up.

**Conclusion:**

In the hands of an experienced surgeon, the routine use of 2.5× magnifying loupes did not reduce the incidence of transient postoperative hypocalcemia after total thyroidectomy with central neck dissection. However, their use was associated with longer operative time and subjectively improved precision, suggesting potential value as a training tool for promoting meticulous dissection technique in less experienced surgeons.

## Introduction

Papillary thyroid carcinoma (PTC) accounts for over 80% of all malignant thyroid tumors, and the standard surgical treatment in the absence of clinically detectable lymph node metastases is total thyroidectomy with prophylactic central neck dissection (level VI) (1, 2). Despite technical advances, postoperative parathyroid insufficiency (HP) remains the most common and most significant complication. Transient hypocalcemia (HC) occurs in 20–40% of patients, and permanent HP in 1–3%, leading to lifelong calcium and vitamin D supplementation, reduced quality of life, and substantial healthcare costs (2).

The primary mechanism of HP is inadvertent ischemia or excision of the parathyroid glands during dissection around the inferior thyroid artery and its branches. Parathyroid glands are extremely sensitive to microvascular injury due to their rich capillary network (up to 30% endothelial cells) and the short half-life of PTH (3–5 minutes), resulting in rapid hormone depletion even during transient ischemia (3). Although systematic identification of all four parathyroid glands is recommended (4), some studies paradoxically suggest that overly aggressive attempts at full visualization may increase the risk of devascularization (4–10).

Advanced visualization technologies, such as near-infrared autofluorescence (NIRAF) and indocyanine green (ICG) angiography, improve intraoperative parathyroid identification and perfusion assessment (11–13), with meta-analyses showing a 45–52% reduction in HC in high-volume centers (13). However, these methods are expensive, technically demanding, and not widely available in routine practice.

Magnifying loupes (typically 2.5×) represent a low-cost and widely accessible alternative that may enhance anatomical detail and promote more careful dissection. However, high-level evidence from randomized controlled trials evaluating loupes in oncologic thyroid surgery, particularly in the hands of experienced surgeons, remains limited.

A review of the specialized literature highlights the most important aspects of the operation and numerous surgical and non-surgical techniques that influence the reduction of postoperative HP risk. A substantial portion of these studies concerns the assessment of the effectiveness of intraoperative use of ML. Although ML are widely recognized as useful tools, the view of their exceptional efficacy is still questioned.

The uniqueness of the presented study lies in the attempt to provide maximum objectification of the assessment. Diagnosis and treatment were conducted in the same highly specialized institution. The operation was performed by the same experienced surgeon with a fixed team. Homogeneity of the compared patient groups was ensured, and patients were excluded from evaluation in whom factors unrelated to the surgical technique itself could affect postoperative parathyroid function.

## Materials and methods

### Study design and patients

The study was conducted as a prospective, randomized, controlled trial in a high-volume referral center for thyroid surgery (Department of General Surgery, Ministry of the Interior and Administration Hospital, Gdańsk) between 2023 and 2025.

To minimize the influence of factors independent of the intervention (tumor histology, stage, risk of parathyroid infiltration, thyroid function status), very strict inclusion criteria were applied.

Initially, 120 patients aged 20–40 years with a preoperative diagnosis of exclusively papillary thyroid carcinoma (PTC, Bethesda V/VI) with T1N0M0 features, without hormonal activity disorders and without treatment for other diseases were qualified. Older patients, in whom microvascular quality could affect postoperative parathyroid function, were excluded. Due to the potential extent of manipulation, only patients with a single tumor up to 2 cm were qualified for the study. Larger lesions, whose cytokine activity could affect circulation and potentially increase the extent of manipulation, were excluded.

Randomization was performed in a 1:1 ratio using sealed, opaque envelopes (concealed allocation), ensuring concealment of assignment until group allocation after obtaining informed consent from patients:

- Loupes group (ML T + 6L): n = 60
- Control group (T + 6L): n = 60

### Study endpoints

The primary endpoint of this study was the incidence of transient postoperative hypocalcemia, defined as serum calcium level < 8.0 mg/dL on the second postoperative day and/or the need for calcium supplementation (oral or intravenous).

Permanent hypoparathyroidism was defined as the need for calcium and/or active vitamin D supplementation persisting for more than 6 months after surgery. All patients were followed up for at least 6 months to assess for permanent hypoparathyroidism.

Secondary endpoints included the number of intraoperatively identified parathyroid glands, the rate of incidental parathyroid excision confirmed by histopathological examination, biochemical changes in serum calcium (ΔCa) and parathyroid hormone (ΔPTH) on postoperative day 2, and operative time.

Exploratory endpoints comprised the incidence and severity of clinical symptoms of hypocalcemia and the need for intravenous calcium supplementation.

Postoperatively, an additional 12 patients were excluded from the final analysis for the following reasons:

- in 5 patients, intraoperative bleeding occurred during dissection, and the intensity of hemostasis-related maneuvers could have influenced postoperative operative field edema,
- in 2 patients, intraoperative involvement of lateral compartment lymph nodes was found, increasing the extent of surgery,
- in 3 patients, postoperative calcium and PTH measurements were repeated, undermining measurement reliability,
- in 2 patients, cardiac rhythm disturbances requiring pharmacological treatment occurred.

Ultimately, 108 patients were included in the final evaluation:

- Loupes group (ML T + 6L): n = 54 (43 females, 11 males)
- Control group (T + 6L): n = 54 (45 females, 9 males)

Group homogeneity was confirmed with respect to mean primary tumor size (2.1 ± 0.6 cm vs 2.2 ± 0.5 cm; p = 0.68).

### Intervention

Ultimately qualified patients underwent total thyroidectomy with prophylactic level VI central neck dissection (T + 6L). All procedures were performed by the same expert in thyroid surgery with a fixed team using a standardized capsular dissection technique. Intraoperative neuromonitoring (IONM) of the recurrent laryngeal nerves was used in all cases. To minimize the risk of thermal or mechanical injury to sensitive structures such as the parathyroid glands and recurrent laryngeal nerves, energy devices were not used in their immediate vicinity; instead, ligation with ties was performed. Bipolar forceps and BiClamp were used selectively only in areas distant from these structures. Parathyroid glands identified intraoperatively were preserved *in situ* with their vascular pedicle whenever possible. No parathyroid autotransplantation was required in any patient.

In the loupes group (ML T + 6L), surgery was performed using 2.5× magnifying loupes (Univet AIR-X 450 model without integrated LED illumination) under standard operating room lighting.

Assessed parameters Intraoperative criteria were:

- number of identified parathyroid glands
- incidence of incidental parathyroid excision
- operative time

All of the above were compared between the loupes group and the control group.

Postoperative criteria were:

- incidence of reported clinical symptoms of HP
- parameters of transient postoperative HC
- changes in serum calcium and PTH levels on postoperative day 2 (ΔCa and ΔPTH)

Calcium and PTH levels were measured in one certified laboratory (using the same method throughout the study), ensuring consistency and minimizing analytical variability.

Hospital length of stay was also recorded.

### Statistical analysis

All statistical analyses were performed using Statistica version 12.5 (StatSoft).

## Results

**Figure 1:**
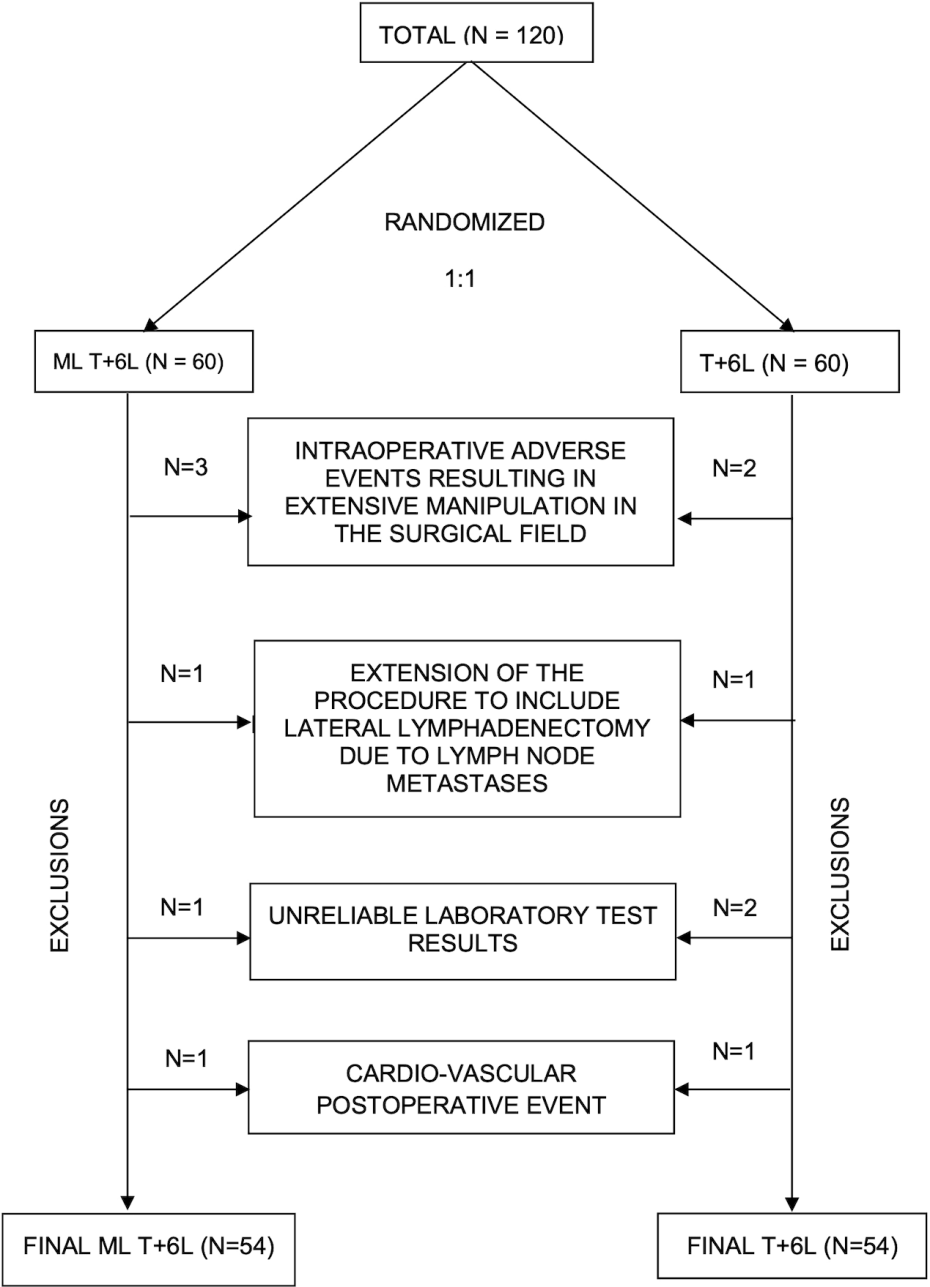
Patient qualification for the study after accounting for variables that could have influenced the result.

A total of 120 patients were assessed for eligibility between 2023 and 2025. After screening and application of inclusion/exclusion criteria, as well as exclusion of 12 patients due to predefined intraoperative and postoperative confounding factors, 108 patients were included in the final analysis (**Figure 1**). Patients were randomly allocated in a 1:1 ratio to the loupes group (ML T + 6L, n = 54) or the control group (T + 6L, n = 54). Baseline characteristics were well balanced between the groups (**Table 1**).

**Table 1:** Baseline characteristics of the study groups.

Parameter	ML T + 6L (n=54)	T+6L (n=54)	p-value*
Age (years, mean ± SD)	39.4 ± 7.3	40.3 ± 9.6	0.842
Female sex, n (%)	43 (79.6)	45 (83.3)	0.805
Preoperative calcium (mg/dL, mean ± SD)	9.47 ± 0.38	9.53 ± 0.36	0.350
Preoperative PTH (pmol/L, mean ± SD)	34.79 ± 11.70	38.7 ± 17.7	0.299

*All p-values from Mann–Whitney U test.

The primary endpoint — incidence of transient postoperative hypocalcemia — did not differ significantly between the groups: 31.5% (17/54) in the loupes group versus 33.3% (18/54) in the control group (p = 1.000).

Clinical symptoms of hypocalcemia were infrequent and comparable between groups. Oral calcium supplementation was required in 31.5% versus 33.3% of patients (p = 1.000), while intravenous calcium supplementation was needed in 5.6% versus 5.6% (p = 1.000). No severe or prolonged symptoms of tetany were observed.

With regard to secondary endpoints, the mean number of intraoperatively identified parathyroid glands was 2.9 ± 1.0 versus 3.2 ± 1.0 (p = 0.080), and the rate of incidental parathyroid excision was identical (24.1% vs 24.1%; p = 1.000). Operative time was significantly longer when magnifying loupes were used (144.1 ± 31.7 min vs 125.4 ± 29.8 min; p = 0.001) (**Table 2**).

**Table 2:** Primary and secondary surgical outcomes.

Parameter	ML T + 6L (n=54)	T+6L (n=54)	p-value*
Primary outcome			
Transient hypocalcemia, n (%)	17 (31.5)	18 (33.3)	1.000
Secondary outcomes			
Parathyroid glands identified (mean ± SD)	2.9 ± 1.0	3.2 ± 1.0	0.080
Incidental parathyroid excision, n (%)	13 (24.1)	13 (24.1)	1.000
Calcium supplementation po, n (%)	17 (31.5)	18 (33.3)	1.000
Calcium supplementation iv, n (%)	3 (5.6)	3 (5.6)	1.000
Hospital stay (days, mean ± SD)	4.3 ± 0.7	4.5 ± 0.8	0.265

*All p-values from Mann–Whitney U test.

Biochemical parameters on the second postoperative day are shown in **Table 3**. The mean decline in serum calcium (ΔCa) was –1.29 ± 0.91 mg/dL in the loupes group versus –1.31 ± 0.86 mg/dL in the control group (p = 0.917). The change in parathyroid hormone (ΔPTH) was –10.1 ± 19.3 pmol/L versus –15.0 ± 19.1 pmol/L (p = 0.163). No patient in either group developed permanent hypoparathyroidism during the minimum 6-month follow-up.

**Table 3:** Biochemical parameters and changes on postoperative day 2.

Parameter	MLT+6L (n=54)	T+6L (n=54)	p-value*
Calcium level (mg/dL, mean ± SD)	8.22 ± 0.81	8.17 ± 0.83	0.754
Δ Calcium (mg/dL, mean ± SD)	–1.29 ± 0.91	–1.31 ± 0.86	0.917
PTH level (pmol/L, mean ± SD)	24.7 ± 19.8	23.8 ± 17.6	0.988
Δ PTH (pmol/L, mean ± SD)	–10.1 ± 19.3	–15.0 ± 19.1	0.163

*All p-values from Mann–Whitney U test.

## Discussion

The main objective of this study was to objectively verify whether 2.5× magnifying loupes – used by an experienced surgeon – provide a real, measurable clinical benefit in parathyroid protection during total thyroidectomy with level VI central neck dissection in papillary thyroid carcinoma. We exposed 54 patients to surgery without loupes to check, under controlled conditions, whether adding a simple visualization tool actually improves outcomes compared to classic naked-eye technique – which is crucial for assessing whether this method should be routinely introduced in everyday oncologic practice.

The history of thyroid surgery shows that since Kocher’s time (late 19th century), the pursuit of precise dissection and parathyroid preservation has been one of the most important challenges. Despite tremendous technical progress over the last 130 years (neuromonitoring, NIRAF, ICG, autofluorescence), the incidence of postoperative transient hypocalcemia still ranges from 20–40%, and permanent hypoparathyroidism occurs in 1–3% of patients (1, 2, 6, 14–17). The consequences of parathyroid insufficiency can be dramatic – from mild paresthesia and tingling, through tetany attacks, heart failure, to rare but potentially fatal complications (e.g., ventricular arrhythmias, pulmonary edema, respiratory failure) (18, 19). Even occasional, transient symptoms in the first postoperative day (tingling, muscle cramps) are highly distressing for many patients – they evoke fear, a sense of life threat, and reduce comfort of life, often for many months (20, 21). Therefore, any method that could significantly reduce this risk – even by a few percent – deserves thorough evaluation.

This work is important precisely because it provides one of the few prospective, randomized studies evaluating loupes in a strictly defined group of PTC patients, under the conditions of a single experienced operator and a single center. Unlike most previous reports, which are retrospective, multicenter, heterogeneous in histology or operator experience, our study was designed to maximally limit confounding factors and provide a “clean” picture of the effect of loupes on outcomes. It is this methodological rigor that justifies why reviewers should invest time in analyzing this paper.

A limitation of the study is the lack of expensive and advanced imaging methods (NIRAF, ICG), which in meta-analyses show a 45–52% reduction in hypocalcemia (13, 22, 23). However, these technologies are costly (system cost >50,000 USD), technically demanding, require specialized equipment, and are not (and likely will not be in the coming years) routinely used in most centers worldwide (24, 25). Therefore, in clinical practice, simple methods still dominate – capsular dissection according to Kocher’s principles and his successors, optionally supported by loupes or headlight.

Magnifying loupes (2.5×) are evaluated ambivalently in the literature. Some authors emphasize their advantages: better identification of anatomical structures, fewer incidental parathyroid excisions, and improved surgeon comfort (26–29). Other studies, especially those conducted by experienced operators, do not show a significant difference in hypocalcemia incidence or number of identified glands (30–33). The drawback of most of these works is the small number of patients (often <50 per group), multicenter design (variable operator experience), heterogeneity of diagnoses (PTC + benign tumors + medullary carcinoma + Graves) or lack of a strict dissection protocol (34, 35).

Our study was designed to eliminate these flaws. A homogeneous group (exclusively PTC T1–T2, single operator, single technique, single assistant, single laboratory) allows for a “clean” assessment of the effect of loupes on parathyroid preservation. The result is clear: loupes did not reduce the incidence of hypocalcemia nor improve intraoperative parathyroid identification. The only significant difference was longer operative time in the loupes group (144 vs 125 min; p=0.001), most likely reflecting the forced slower, more cautious pace of work.

Interpretation of this result is straightforward: in the hands of an experienced surgeon, loupes do not provide additional clinical benefit – manual technique and anatomical knowledge remain decisive. However, it is precisely this forced slowing and greater attention to detail that may represent the greatest value of loupes – as an educational tool. Young surgeons, residents, and less experienced operators learn from this slower pace to respect anatomical planes, avoid haste, and protect parathyroid vessels (36–39). It is this behavioral change – not the mere number of visualized glands – that may ultimately bring the greatest benefit to patients in the long term.

Future studies should evaluate loupes in residency and multicenter settings, including the learning curve, subjective precision assessment by operators, and long-term costs of parathyroid-related complications. A hybrid approach (loupes + selective use of NIRAF/ICG in difficult cases) may be the most rational solution in everyday practice.

## Limitations

This study has several limitations that should be acknowledged. First, the trial was not prospectively registered in a public clinical trial registry such as ClinicalTrials.gov. The compared interventions (use of 2.5× magnifying loupes versus naked-eye surgery) represent long-established surgical practices that have been used worldwide for decades.

Second, although the incidence of transient postoperative hypocalcemia was the main clinical outcome of interest from the conceptual stage of the study, it was not formally predefined as the single primary endpoint before recruitment began, and no formal sample size calculation based on this endpoint was performed.

Third, 12 patients were excluded after randomization due to intraoperative or postoperative events that could confound parathyroid function (significant bleeding during dissection, unexpected extension of surgery due to lateral lymph node involvement, unreliable laboratory measurements, or cardiac rhythm disturbances). Although the number of exclusions was small and balanced between the two groups, an intention-to-treat analysis was not conducted.

Furthermore, the study population was highly selected (patients aged 20–40 years with low-risk papillary thyroid carcinoma, tumor size ≤ 2 cm, and no parathyroid infiltration), and all procedures were performed by a single experienced surgeon in a high-volume center. Consequently, the generalizability of the findings to routine clinical practice, low-volume centers, or less experienced surgeons is limited.

Finally, the suggestion that magnifying loupes may be particularly beneficial as a training tool for young surgeons is based primarily on the authors’ clinical experience and subjective observations of improved tissue handling rather than objective measurements of learning curves or trainee performance. Future studies should evaluate this aspect more systematically.

Despite these limitations, the present study provides valuable randomized data on two standard surgical techniques in a well-defined and homogeneous cohort with complete follow-up.

## Conclusion

In this prospective randomized controlled trial, the use of 2.5× magnifying loupes did not significantly reduce the incidence of transient postoperative hypocalcemia compared with naked-eye surgery when total thyroidectomy with central neck dissection (T + 6L) was performed by an experienced surgeon. No differences were observed in the primary endpoint or in secondary clinical and biochemical outcomes.

Nevertheless, magnifying loupes were associated with a longer operative time and subjectively improved precision and deliberate tissue handling. These findings suggest that loupes may promote greater surgical mindfulness and meticulous dissection technique – factors considered important for preserving parathyroid vascular integrity.

Magnifying loupes remain a simple and low-cost tool. While the present study does not provide direct evidence of their educational benefit, they may have potential value in surgical training by promoting careful and unhurried dissection technique in less experienced surgeons. Future studies should objectively evaluate the impact of loupe magnification on the learning curve and performance of trainees in thyroid surgery.

## Data Availability

The raw data supporting the conclusions of this article will be made available by the authors, without undue reservation.
